# NKCC1, an Elusive Molecular Target in Brain Development: Making Sense of the Existing Data

**DOI:** 10.3390/cells9122607

**Published:** 2020-12-04

**Authors:** Mari A. Virtanen, Pavel Uvarov, Christian A. Hübner, Kai Kaila

**Affiliations:** 1 Molecular and Integrative Biosciences, University of Helsinki, 00014 Helsinki, Finland; mari.virtanen@helsinki.fi (M.A.V.); pavel.uvarov@helsinki.fi (P.U.); 2 Neuroscience Center, Helsinki Institute of Life Science, University of Helsinki, 00014 Helsinki, Finland; 3 Institute of Human Genetics, Jena University Hospital, Friedrich Schiller Universität, 07747 Jena, Germany; Christian.Huebner@med.uni-jena.de

**Keywords:** *Slc12a2*, ion regulation, chloride, GABA, epilepsy, NKCC1, KCC2

## Abstract

Ionotropic GABA transmission is mediated by anion (mainly Cl^−^)-permeable GABA_A_ receptors (GABA_A_Rs). In immature neurons, GABA exerts depolarizing and sometimes functionally excitatory actions, based on active uptake of Cl^−^ by the Na-K-2Cl cotransporter NKCC1. While functional evidence firmly shows NKCC1-mediated ion transport in immature and diseased neurons, molecular detection of NKCC1 in the brain has turned out to be extremely difficult. In this review, we describe the highly inconsistent data that are available on the cell type-specific expression patterns of the NKCC1 mRNA and protein in the CNS. We discuss the major technical caveats, including a lack of knock-out-controlled immunohistochemistry in the forebrain, possible effects of alternative splicing on the binding of antibodies and RNA probes, and the wide expression of NKCC1 in different cell types, which make whole-tissue analyses of NKCC1 useless for studying its neuronal expression. We also review novel single-cell RNAseq data showing that most of the NKCC1 in the adult CNS may, in fact, be expressed in non-neuronal cells, especially in glia. As future directions, we suggest single-cell NKCC1 mRNA and protein analyses and the use of genetically tagged endogenous proteins or systematically designed novel antibodies, together with proper knock-out controls, for the visualization of endogenous NKCC1 in distinct brain cell types and their subcellular compartments.

## 1. Introduction

NKCC1, encoded by *Slc12a2*, belongs to the SLC12 family of cation-chloride cotransporters (CCCs), which also includes the Na-K-2Cl cotransporter 2 (NKCC2), the Na-Cl cotransporter (NCC), the K-Cl cotransporters 1–4 (KCC1-4), and two orphan members, CCC9 and CIP1, with largely unknown physiological roles. For many of the CCC members, cell-specific expression patterns have been described (for review, see [1]). NKCC2 and NCC, for instance, are predominantly found in the kidney [2], where they mediate salt reabsorption. Only one of the CCC members, KCC2, is known to be a neuron-specific transporter in the central nervous system (CNS) [3,4]. The role of KCC2 as the major neuronal Cl^−^ extruder and its other functions have been described in several reviews [5,6,7].

Among neuroscientists, NKCC1 is best known for its role in maintaining a high intracellular Cl^−^ concentration in immature neurons. During the second postnatal week in rats and mice [4] and around the time of full-term birth in humans [8,9], KCC2 is upregulated, leading to a decrease in neuronal intracellular chloride concentration ([Cl^−^]_i_) and a shift towards more hyperpolarizing GABAergic responses. NKCC1 is currently gaining increased attention as a possible therapeutic target for treating a myriad of CNS disorders [10], and the search for brain-permeable and specific NKCC1 drugs is heating up [11,12,13]. However, a major caveat here is that our understanding of the spatiotemporal expression patterns of NKCC1 in the brain is still in its infancy.

Indeed, emerging evidence indicates that glial expression of NKCC1 in the mature brain is much more important than previously thought. For example, a recent functional study has shown that long-term potentiation (LTP) of glutamatergic synapses in the mouse and rat hippocampus induced an NKCC1-dependent withdrawal of perisynaptic astroglial processes, which boosts extrasynaptic glutamate escape, leading to enhanced inter-synaptic cross-talk [14]. Thus, glial NKCC1 seems to regulate neuronal signaling and plasticity in robust and unexpected ways. Furthermore, NKCC1 in non-neuronal cells can control brain physiology at the whole-tissue level, as seen in ischemia-induced brain edema [15,16], blood–brain barrier disruption [17], and astrocytic swelling [18].

In this review, we give a short summary of the various functions of NKCC1 in different tissues. More specifically, we address the question which cell types in the CNS express NKCC1 and how this expression is regulated during development, by going, in a systematic manner, through the pertinent literature on NKCC1 protein and mRNA expression in the CNS. We compare, in detail, the antibodies and probes used in different studies, and we demonstrate that the inconsistent results are not solely explained by the probes or antibodies used. In addition to the traditional immunohistochemical and in situ hybridization studies, we discuss new data that are available in single-cell RNAseq databases showing that NKCC1 expression in certain glial subtypes is very high—in fact, much higher than in neurons. In conclusion, we call for future studies employing novel single-cell methods for exploring cellular NKCC1 expression patterns in the developing and mature CNS.

## 2. Molecular Structure of NKCC1

**Figure 1:**
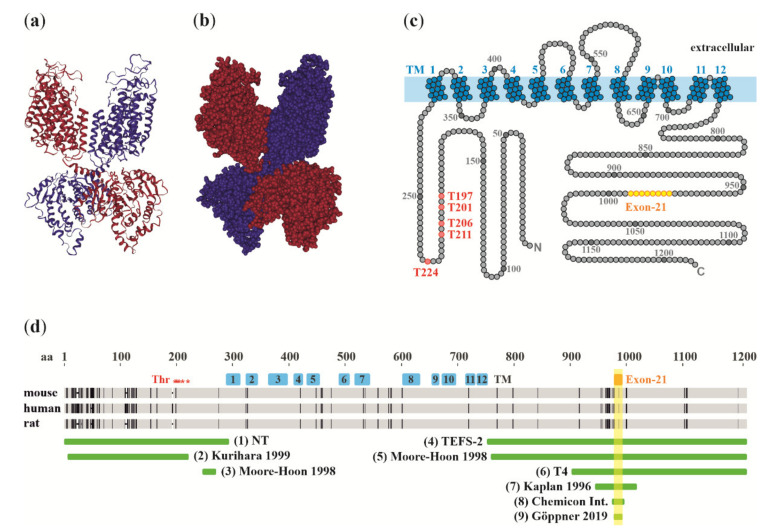
NKCC1 protein structure, and location of antigens utilized in the generation of antibodies for protein analyses. Cryogenic electron microscopy (CryoEM) structure of the NKCC1 dimer shown as a 3D ribbon model (**a**) and a space-filling model (**b**), colored by subunit (red, blue). The C-terminal domain of one protomer is located beneath the transmembrane domain of the other protomer, producing a domain-swapped architecture. Data extracted from Chew et al., 2019, RCSB PDB [25] using NGL viewer [26]. (**c**) Schematic representation of NKCC1 structure showing the 12 transmembrane domains and intracellular N- and C-terminal domains. Phosphorylation sites are indicated in red, and the alternatively spliced region encoded by exon 21 is highlighted in yellow. (**d**) NKCC1 regions used for producing the antibodies (green). (1) Affinity-purified rabbit antibody produced against the entire N-terminus of human NKCC1 [27], (2) polyclonal rabbit antibody raised against amino acids 3–202 of rat NKCC1 [28], (3) α-NT, rabbit polyclonal antibody against aa 238–261 or rat NKCC1 [29], (4) TEFS-2, affinity-purified rabbit polyclonal antibody produced against the entire C-terminus of human NKCC1 (characterized in [30]), (5) α-wCT, rabbit polyclonal antibody, aa 750–1203 of rat NKCC1 [29], (6) T4, mouse monoclonal antibody, aa 902–1212 of human NKCC1 [31], (7) rabbit polyclonal antibody, aa 938–1011 of mouse NKCC1 [32], (8) Chemicon International, affinity-purified rabbit polyclonal antibody, aa 967–988 of mouse NKCC1 [29,33], (9) affinity-purified rabbit polyclonal antibody raised against aa 977–991 of mouse NKCC1 [34]. Linear protein sequences of mouse, human, and rat NKCC1 are aligned and depicted in gray, with mismatches shown by vertical lines and gaps as dashes. Predicted transmembrane domains are indicated in blue, and the region encoded by exon 21 is highlighted in yellow. The five well-studied phosphorylation sites (Thr197, Thr201, Thr206, Thr211, and Thr224) are indicated by asterisks.

NKCC1 mediates a tightly coupled electroneutral uptake of Na^+^, K^+^, and Cl^−^ ions with a stoichiometry of 1:1:2. The monomer, two of which are required for the dimeric functional transporter [19], consists of twelve transmembrane segments flanked by intracellular N-terminal and C-terminal domains [20,21,22]. Recently, single-particle electron cryo-microscopy has confirmed the predicted dimeric structure and allowed detailed exploration of functionally important secondary and tertiary structures, including the ion translocation pore, extracellular cap domain, and the surface residues forming the interface between dimer subunits [23,24] (Figure 1a–c).

The large C-terminal domain of NKCC1 contains a 16-amino acid fragment encoded by exon 21, which can be spliced alternatively. This gives rise to two splice variants, NKCC1a and NKCC1b, that are otherwise similar but whereof the latter is devoid of the 16-amino acid fragment [35]. Although the physiological significance of the two splice variants is still unclear, the exon 21-encoded region contains two evolutionarily conserved regulatory motifs: a putative consensus sequence for PKA phosphorylation [35] and a dileucine motif. The latter is thought to constitute the smallest essential component of the basolateral sorting signal of NKCC1 in polarized epithelial cells [36]. Indeed, NKCC1 is usually localized in the basolateral membrane [37], but a notable exception is the choroid plexus epithelium, where NKCC1 is highly expressed in the apical membrane [38,39].

## 3. NKCC1 Functions outside the CNS

In contrast to the CNS neuron-specific KCC2, NKCC1 shows a wide distribution in a variety of tissues and cell types within and outside the nervous system, similar to KCC3 [40,41]. Accordingly, NKCC1-mediated Cl^−^ transport serves numerous physiological functions in adult animals (reviewed in [42]). In smooth muscle cells, NKCC1 facilitates contraction by maintaining a depolarizing driving force for currents across Cl^−^-permeable channels [43,44]. NKCC1 also participates in Cl^−^-based fluid secretion in exocrine glands, such as salivary, sweat, lacrimal, and pancreatic glands, as well as in the lung, gastric, intestinal, and renal epithelia [42]. In the inner ear, NKCC1 is involved in K^+^-driven secretion of the endolymph [45]. In the zona glomerulosa cells of the adrenal gland, the depolarizing driving force for Cl^−^ currents via ClC-2 channels facilitates the secretion of aldosterone [34]. In many cell types, NKCC1-mediated Cl^−^ transport is a mechanism of cell volume regulation [46], and NKCC1-mediated swelling precedes mitotic division in virtually all cells [47].

Furthermore, NKCC1 is emerging as an important regulator of several major signaling pathways involved in cell mass regulation [48]. In HEK293T and HeLa cells, and in mouse colonic epithelia, endogenous NKCC1 inhibits Leu transporter LAT1, as well as PI3K/Akt and Erk signaling, leading to suppressed activation of the mechanistic target of rapamycin complex 1 (mTORC1) which is a master regulator of energy metabolism. Thus, NKCC1 might provide a long-sought link connecting cell volume and cell mass regulation, through NKCC1-mediated ion transport and mTORC1 activation, respectively.

Several constitutive NKCC1 knock-out (NKCC1-KO) mouse models have been generated by targeting exon 9 [49], exon 6 [50], exon 9, 10, and 11 [51], or exon 24 and 25 [51], as well as exon 8, 9, and 10 [52], and by identification of a spontaneous mutant with a premature stop codon in exon 17 [52]. NKCC1-KO mice are viable, likely due to strong developmental compensation (for a review on biological robustness, see [53]), and they have been reported to suffer from inner ear defects, including deafness and imbalance [49,50,51,52,54], growth retardation [50,51], gastrointestinal deficits [50], reduced blood pressure [50], infertility [51], and decreased sensitivity to pain [55,56]. Furthermore, a radiation-induced NKCC1 mutant mouse, *Shaker-with-syndactylism*, carries a point mutation (nt2955 ins(A)) in exon 21, causing a frameshift and a subsequent premature termination codon in the NKCC1a splice variant only [54]. Since exon 21 is not present in the NKCC1b mRNA, the mutation does not affect the translation of the NKCC1b splice variant, and thus this mouse line can be considered an NKCC1a-specific KO. The *shaker-with-syndactylism* homozygotes are deaf and exhibit vestibular dysfunction [57,58]. So far, several human mutations of NKCC1 have been reported [59,60,61,62,63,64]. Patients with deletion in NKCC1 suffer from multiorgan failure [60] or global developmental delay, together with hearing loss, gastrointestinal abnormalities, and absent salivation (named Kilquist syndrome [59]), while a gain-of-function missense variant of NKCC1 has been linked to schizophrenia [62].

**Figure 2:**
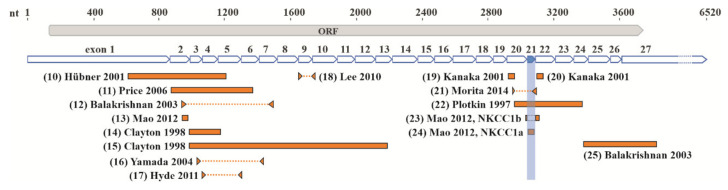
Regions of NKCC1 mRNA targeted in the expression studies. Regions of NKCC1 mRNA targeted by in situ probes (orange rectangles) or PCR amplification (orange dashed lines). Mouse NKCC1 nucleotide sequence NM_009194 is used as a reference sequence for mouse in situ probes and PCR fragments. For rat and human in situ probes and PCR fragments, the corresponding mouse nucleotide sequences are indicated. (10) Mouse in situ probe nt 615–1206 [68], (11) rat in situ probe nt 883–1376 [66], (12) rat PCR amplicon nt 938–1468 [69], (13) rat in situ probe nt 946–983 [65], (14, 15) rat in situ probes 988–1179 and nt 988–2190 of rat NKCC1 [70], (16) rat PCR fragment nt 1033–1435 amplified with single-cell RT-PCR [71], (17) exon 4 and 5 of human NKCC1 targeted with qRT-PCR [72], (18) rat amplicon nt 1650–1751 amplified by RT-PCR [73], (19, 20) rat in situ probes against nt 2925–2960 and nt 3099–3134 [74], (21) human PCR fragment nt 2950–3093 [75], (22) mouse in situ probe against nt 2951–3365 [38], (23, 24) rat NKCC1a and NKCC1b isoforms-specific in situ probes against nucleotides 3041–3073 and 3024–3040 + 3089–3103, respectively [65], (25) rat in situ probe against nt 3373–3814 rat NKCC1 [69]. Part of the 3’UTR encoded in exon 27 is omitted for clarity (dashed line).

In the peripheral nervous system, knock-out-controlled NKCC1 immunoreactivity has been reported, outlining nearly all somata of the primary sensory neurons in dorsal root ganglia (DRG) [55] (rabbit polyclonal antibody by Kaplan, 1996, antigen (7) in Figure 1), [65] (polyclonal rabbit antibodies by Kurihara, 1999, Moore-Hoon and Turner, 1998, and Kaplan, 1996, antigens (2), (5), and (7), respectively). However, this staining pattern has been suggested to originate at least partially from satellite glia, which tightly surround the DRG neurons [66] (affinity-purified rabbit polyclonal antibodies by McDaniel and Lytle, 1999, and Del Castillo, 2005, antigens (1) and (4), respectively, and nucleotide (11) in Figure 2), ref. [67] (T4, antigen (6) in Figure 1).

In contrast to non-neuronal tissues, such as the choroid plexus [38] and the endolymph-secreting stria vascularis of the inner ear [52,76], where a strong NKCC1 signal can be relatively easily detected even at the subcellular level using immunohistochemistry, localizing NKCC1 in neuronal and glial cells of the brain has turned out to be extremely difficult. It is currently unclear whether this could be due to differences in expression levels, or if there are technical issues specifically hindering the detection of NKCC1 in the CNS, such as epitope masking by interacting proteins or post-translational modifications, or alternative splicing affecting the targeted epitopes.

## 4. Functional Data Show Cl^−^ Uptake by NKCC1 in CNS Neurons

In central neurons, uptake of Cl^−^ by NKCC1 has been implicated in proliferation and cell cycle regulation [77,78,79], as well as in programmed cell death of neocortical Cajal–Retzius neurons [80]. With regard to the functions of NKCC1 in controlling GABAergic transmission, most of the available data are based on recordings in pyramidal neurons during early postnatal development in rats and mice. This work has demonstrated that NKCC1 maintains a high [Cl^−^]_i_, and a Cl^−^ equilibrium potential (E_Cl_) which sets the reversal potential of GABA_A_R-mediated currents (E_GABA_) to a much more positive level than the resting potential, thus promoting depolarizing and sometimes functionally excitatory GABA_A_R responses [71,81,82,83].

When using electrophysiological or other approaches for the identification of NKCC1 activity, it is important to note that neurons have additional ion transporters which take up chloride, such as the Cl^−^/HCO_3_^−^ exchangers *Slc4a3* [84] and *Slc26a11* [85,86]. Furthermore, a depolarization caused by GABA_A_Rs is not sufficient evidence for the presence of NKCC1 or, in fact, of any active Cl^−^ uptake mechanism. This is because GABA_A_Rs show a significant permeability to HCO_3_^−^, an ion that is accumulated into neurons by pH regulatory mechanisms (reviewed in [87,88]). Thus, opening of GABA_A_R channels in neurons with a low [Cl^−^]_i_, such as neocortical neurons at rest, will lead to a significant depolarization caused by the inwardly directed HCO_3_^−^ current, despite the presence of active K-Cl cotransport (E_HCO3_ > E_GABA_ > E_Cl_) [89]. While this mechanism has received much less attention in the current literature than what was the case after the original identification [90], its neurophysiological impact [91] has obviously not changed.

In electrophysiological work on depolarizing GABA_A_R responses, the specific NKCC1 blocker, bumetanide, has been used as a very effective pharmacological tool [10,71,81,92,93,94]. Unfortunately, because of its extremely poor pharmacokinetic properties (including low permeability across the blood–brain barrier and 97–98% binding to plasma proteins [95,96,97,98,99]), the use of this drug is limited to in vitro preparations, and to in vivo experiments in which the drug can be directly applied to the target area in the CNS (for review, see [99,100]).

The first direct in vivo evidence of active Cl^−^ accumulation by NKCC1 in developing pyramidal neurons comes from a recent study that used Cl^−^ imaging with a newly developed Cl^−^ and pH sensor, LSSmClopHensor [93]. In postnatal (P) day P4–P5 mouse cortical neurons, direct application of bumetanide in the brain produced a substantial decrease in [Cl^−^]_i_, as seen when monitoring individual neurons, indicating that NKCC1 maintains a high [Cl^−^]_i_ in developing pyramidal neurons under in vivo conditions [93]. In agreement with this, [Cl^−^]_i_ was much lower at P18–P51 than P4–P5. While the above differences related to bumetanide and maturational stage are robust, the relatively high absolute levels of Cl^−^ in these experiments are likely to be attributable to the ongoing activity in the neurons in vivo and to possible technical problems in calibration. Furthermore, the first evidence for functionally excitatory actions of GABAergic interneurons in the immature hippocampus in vivo has recently been published [101]. Similar to the neurons of the immature brain, NKCC1 maintains a high [Cl^−^]_i_ in neuronal progenitors of the adult subventricular zone [102,103] and dentate gyrus [104].

In contrast to the above, little is known about NKCC1 functions in adult CNS neurons. In horizontal cells of the adult mouse retina, NKCC1 maintains a depolarized E_GABA_ value, and horizontal cell depolarization caused by their own GABA release affects photoreceptor cone voltage-gated Ca^2+^ channels through pH regulation of the synaptic cleft [105]. An important and often neglected finding is that NKCC1 can drive a GABA_A_R-mediated depolarization of presynaptic glutamatergic terminals in hippocampal CA3 neurons [106], in the hypothalamus [107], and in parallel fibers in the cerebellum [108]. Electrophysiological data also indicate that in mature pyramidal neurons, NKCC1 is present in the axon initial segment (AIS), which is the only target of axo-axonic (chandelier) interneurons, where it sustains a depolarized E_GABA_ value [109]. Indeed, axo-axonic interneurons have been reported to depolarize, and perhaps even to excite, mature cortical pyramidal neurons [110,111]. However, conflicting results have been presented showing hyperpolarizing GABAergic responses along the whole neuronal axis including the AIS [112,113]. These discrepancies were suggested to be reconciled by a substantially delayed shift to hyperpolarizing responses at the AIS around P55 in the mouse prefrontal cortex and by P40 in the mouse somatosensory cortex [114,115]. In general, the targeting of different chloride cotransporters to specific subcellular locations is an intriguing and potentially very important mechanism, as it allows the neuron to modulate inputs from specific subpopulations of interneurons through the spatial subcellular heterogeneity of [Cl^−^]_i_ [109,110,116,117,118]. Moreover, co-expression of two oppositely directed ion pumps would provide a means for the very tight regulation of [Cl^−^]_i_ within a given subcellular microdomain (for a discussion on ion regulatory pumps acting in a push–pull manner, see [119]).

Many of the in vitro findings on depolarizing GABA obtained using bumetanide have been confirmed with NKCC1-KO hippocampal tissue [94,109,120]. However, strong compensation for the lack of Cl^−^ uptake in NKCC1-KO mice seems to be present at the network level [94], similar to the adaptive, compensatory regulation of neuronal excitability reported in mice lacking α6 and δ [121] or α1 subunits [122,123] of the GABA_A_ receptor, or HCN1 channels [124]. Despite the large number of NKCC1-KO mouse lines, it remains unclear whether NKCC1 deficiency restricted to CNS neurons only would result in profound defects in brain functions and behavioral abnormalities. Since constitutive NKCC1-KO gives rise to several serious systemic effects, such as growth retardation and reduced blood pressure, as well as to motor deficits due to inner ear problems, using these animals for assessing the direct effect of NKCC1 deficiency on brain function is problematic if not impossible. Mouse lines with the knock-out of NKCC1 in specific cell types have recently been generated [52,125], and their characterization should help to address the above problem. A doxycycline-controllable NKCC1 knock-down (NKCC1-KD) mouse has also been generated and employed in a study on the role of cochlear NKCC1 in hearing loss [126]. This mouse would, quite obviously, hold great promise also for studying the roles of NKCC1 in CNS neurons.

Unlike most central neurons, peripheral neurons in the DRG and trigeminal ganglia maintain depolarizing GABA_A_R-mediated responses throughout postnatal life [55,127,128]. The depolarizing GABA_A_R responses are reduced in NKCC1-KO animals, indicating that the depolarized E_GABA_ value is maintained by NKCC1 [55]. Furthermore, NKCC1 has been implicated as an important factor in inflammatory and tissue damage pain (reviewed in [5,129,130]).

## 5. NKCC1 Protein Levels and Localization in the CNS

The wide expression of NKCC1 in various cell types poses problems for meaningful quantifications. For instance, brain tissue samples used in Western blots contain NKCC1 molecules originating from various types of neurons and glia, blood vessels, and other epithelial cells, and all information on NKCC1 distribution among specific cell types is lost. Despite these obvious limitations, Western blot data have been widely (and inadequately) used in a large number of publications to back up hypotheses and preconceived ideas on changes in neuronal NKCC1 levels during brain development and damage, and in various kinds of experimental protocols in vivo and in vitro. While single-cell Western blotting [131] holds great promise for tackling this issue, it has not yet been used for studying NKCC1 expression.

Immunohistochemistry is an obvious way, in principle, of studying cell-specific protein expression levels. In the case of NKCC1, multiple antibodies against various peptide antigens have been generated (Figure 1d), but, unfortunately, to the authors’ present best knowledge, there is not a single published NKCC1 antibody that has been firmly demonstrated to yield a fully NKCC1-specific signal in the parenchyma of the adult forebrain. Therefore (and unsurprisingly), despite the large number of the NKCC1 antibodies available, there is no consensus regarding which cell types do or do not express the protein, and contradictory results have been obtained by different groups using even the same antibody (Table 1).

To provide an overall picture of the current status of NKCC1 immunohistochemistry in adult rat and mouse brain tissue, we will focus here on highly cited papers in this field, referring to Figure 1 for the antigen which has been targeted by each antibody. Using a rabbit polyclonal antibody [32] (antigen (7) in Figure 1), Plotkin and colleagues observed purely neuronal immunoreactivity in all cortical layers of the rat cerebral cortex, with staining of neuronal somata and dendrites, but not of (GFAP-labeled) astrocytes [38]. Strong neuronal NKCC1 immunoreactivity, based on the T4 mouse monoclonal antibody [31] (antigen (6) in Figure 1), was also found in dendritic processes of pyramidal neurons in the rat hippocampus [132]. In contrast, other studies performed in adult rats indicate expression also in non-neuronal cells. For instance, in addition to a strong signal observed in dendrites of hippocampal and cortical pyramidal neurons, NKCC1 immunoreactivity for the T4 antibody was detected in GFAP-positive perivascular astrocytic processes in several brain areas and in GFAP-positive cells of the corpus callosum [133] (antigen (6) in Figure 1). Yet another study, using the rabbit polyclonal antibody mentioned above [32], reported NKCC1 staining in somata of both developing and mature rat cortical neurons, but only a transient expression in developing oligodendrocytes, which has its peak around P14 and is undetectable in adults [134] (antigen (7) in Figure 1). Further examples on published immunohistochemistry with putative NKCC1 antibodies are provided in Table 1.

Considering the lack of consensus regarding cell types expressing the NKCC1 protein in the adult forebrain, it is unfortunate that no thorough KO-controlled immunohistochemical analyses have been conducted in these regions. However, KO-controlled data are available for other regions of the CNS. For instance, in the mouse brainstem, subpopulations of neurons and some small-sized glial cells were found to be stained with the T4 antibody (epitope (6) in Figure 1) in the wild type but not in NKCC1-KO mice [135]. In the adult mouse retina, neuronal staining has been reported by two knock-out-controlled studies: one study detected strong labeling in horizontal cells of the outer plexiform layer [136] (rabbit polyclonal antibody by Kaplan, 1996; in Figure 1, antigen (7)), whereas the other study found NKCC1 immunoreactivity not only in horizontal cells, but also in rod-bipolar dendrites and diffuse labeling in photoreceptor terminals [137] (T4, antigen (6) in Figure 1).

Results obtained by immunohistochemistry are highly dependent not only on the properties of the antibodies but also on subtle differences in the protocols used [138,139]. One of the main problems in immunohistochemical detection of NKCC1 seems to be epitope masking during standard paraformaldehyde fixation [65]. Several strategies have been proposed for antigen retrieval, such as treatment with 1% sodium dodecyl sulfate, which is recommended for protocols with the monoclonal T4 antibody [27], or heat-induced epitope retrieval by boiling the sections in sodium citrate solution [66,77]. Considering the various approaches used for NKCC1 immunohistochemistry, parallel experiments on KO tissue are absolutely necessary. This is important not only for controlling the specificity of the antibody, but also of the validity of the whole staining protocol.

Unintended splice isoform specificity or preference has previously been suggested to partly explain the inconsistent results obtained with different NKCC1 antibodies [70,99]. Many of the antigens used to produce NKCC1 antibodies contain the region encoded by exon 21 (antigens 4–9 in Figure 1d), which is alternatively spliced into NKCC1a and NKCC1b isoforms. However, this 16-aa fragment constitutes only a small fraction of the total length of most of the antigens used for producing NKCC1 antibodies (e.g., about 300 amino acids for the widely used T4 antibody). A notable exception is the rabbit polyclonal NKCC1 antibody generated by He and colleagues [33] and subsequently validated by the Turner lab [29], which is currently commercially available (Chemicon International, Cat No. AB3560P). This antibody was raised against a 22-aa peptide, which encompasses the 16 amino acids encoded by exon 21, thus making it, with high probability, specific for the NKCC1a isoform (antigen (8) in Figure 1d). Another exception is a recently published antibody, which targets an epitope encoded by exon 21 only [34] (antigen (9) in Figure 1d). Additional variability in NKCC1 detection by different NKCC1 antibodies may occur due to epitope phosphorylation (for mechanisms of NKCC1 phosphorylation, see [140,141]), or due to sequence diversity between NKCC1 homologues in different species. Most parts of NKCC1 are relatively well conserved between mice, rats, humans, and other mammals (sequence identity is 98% between mice and rats, and 94% between mice and humans), and the discrepancies in amino acid sequences between these species are mainly located in N-terminal parts and C-terminal regions around exon 21 (depicted in Figure 1d by vertical black lines).

## 6. NKCC1 mRNA Expression in the CNS

A complementary strategy to study the NKCC1 expression pattern in the brain at the cellular level is to shift the focus from protein to mRNA detection. This approach cannot be considered as a substitute for immunohistochemical analyses, since the presence of NKCC1 mRNA does not necessarily indicate the expression of the NKCC1 protein. Except for in situ hybridization, most of the previously used methods for NKCC1 mRNA analysis (Northern blot, RNase protection assay, reverse transcription polymerase chain reaction (RT-PCR)) are based on whole-tissue samples. Thus, in a manner similar to Western blots, all cell-specific information is lost and only whole-tissue mRNA levels can be analyzed. However, these techniques can provide information about alternatively spliced NKCC1 transcripts at a low level of structural resolution.

Before the widespread use of single-cell RNA sequencing (scRNAseq), in situ hybridization remained the only method to study NKCC1 mRNA distribution in the brain with cellular resolution. Astonishingly, the results with in situ hybridization, as described below, seem to be as divergent and mutually contradictory as those obtained with immunohistochemical analysis.

In some studies, NKCC1-positive cells with clear neuronal morphology were detected in all cortical layers of the adult rat neocortex [38] (nucleotide (22) in Figure 2), and no signal was observed in areas with high glial density [70] (nucleotides (14) and (15) in Figure 2). However, it is worth pointing out that in the Plotkin et al., 1997, study [38], probes against mouse NKCC1 mRNA were used on rat tissue, which may affect their sensitivity. In contrast, in another study, a strong expression was described in pyramidal cells of the rat hippocampus, whereas in the cortex, expression was weak and mainly detected in small glial-like cells [74] (nucleotides (19) and (20) in Figure 2). A developmental change in NKCC1 mRNA expression from a mainly neuronal pattern at birth to a glial pattern in the adult was reported in various areas of the mouse brain [68] (nucleotide (10) in Figure 2).

Altogether, in the adult forebrain, NKCC1 mRNA expression in either neurons or glia, or both neurons and glia, has been reported. The location of the probes used for in situ hybridization and RNase protection assay, as well as primers for RT-PCR, particularly with respect to the alternatively spliced exon 21, does not seem to give a plausible explanation for the divergent results (Figure 2).

It is also important to note that the absolute number of mRNA molecules per cell may be surprisingly low compared to the number of corresponding protein molecules. In mammalian cell lines, the total protein copy numbers have been estimated to be approximately three–four orders of magnitude higher than the corresponding mRNA numbers [142,143]. This means that even a few mRNA transcripts could give rise to a functionally efficient protein pool through the extensive amplification that occurs during translation. A further level of complexity arises from the RNA localization and local translation in subcellular compartments. In neurons, local translation in dendrites and axons has recently emerged as an important and widespread mechanism of targeted protein production [144,145,146,147]. As suggested in the “sushi belt model” [145], intraneuronal ribonucleoprotein particles (RNPs) circulate in dendrites, like on a conveyor belt in a sushi restaurant, ready to release transcripts in an on-demand manner for local translation of, e.g., plasticity-related proteins in activated synapses. Considering that the mRNA copy numbers in the dendritic tree seem to be particularly low, localization or recruitment in strategically important sites, such as dendritic spines or the pre-synaptic terminal, could allow a functionally sufficient amount of protein to be produced even from just a few mRNA transcripts [148]. As a consequence, for genes that are expressed at low levels, the signal-to-noise ratio in conventional in situ hybridization may be too low, leading to variability in results.

The recent advances in single-cell transcriptomics hold great promise for studying NKCC1 mRNA expression in different cell types. A systematic survey of transcriptomics in distinct cell types has been provided for the adult [149,150,151] as well as for the developing mouse brain [152], and the corresponding datasets are available online [153,154,155,156]. Detailed analysis of these data reveals that NKCC1 mRNA expression is very broad and may vary up to 100-fold among different cell types in the adult brain. In the adult brain parenchyma, the strongest NKCC1 expression was found in oligodendrocytes [153,154,156,157], and specifically in oligodendrocyte precursor cells [153,156], newly formed oligodendrocytes [153,154], and in myelin-forming oligodendrocytes [153,154]. In certain regions, such as the hippocampus, oligodendrocytic NKCC1 expression was even higher than that of the choroid plexus [156], which is well known for its strong NKCC1 expression. Interestingly, NKCC1 has been found to regulate proliferation and maturation of oligodendrocyte precursor cells in the adult mouse cerebellar white matter [125]. Regarding other cell types in the brain, high levels of NKCC1 expression were found in mural cells, fibroblasts, and microglia [156]. In contrast, neuronal NKCC1 expression seems to be low according to data in all available databases, down to 20-fold lower than that of the oligodendrocytes [153,156]. Similarly, NKCC1 expression in the embryonic day 14.5 and P0 mouse cortex was found to be mainly non-neuronal, being highest in endothelial cells and microglia [155]. A major conclusion based on these data is that, in general, NKCC1 expression in glia is much higher than in neurons.

An additional advantage of the single-cell RNAseq approach is the possibility to assess the cellular specificity of NKCC1 splice variants (e.g., NKCC1a and NKCC1b) that may help us to better understand the functions of these isoforms in neuronal and glial cells. Based mainly on RT-PCR and RNase protection experiments, our current knowledge about the relative expression of NKCC1a and NKCC1b is rudimentary and contradictory. Although NKCC1b was initially indicated as the major NKCC1 splice variant in the human brain [158], recent data clearly demonstrate the opposite: NKCC1a levels are about three times higher than NKCC1b in the adult mouse brain [39]. The cellular specificity of the NKCC1a and NKCC1b isoforms is important, since these two isoforms may have different subcellular localizations [36] and post-translational modifications [35], resulting in different functional properties at the levels of membrane trafficking and ion translocation.

## 7. NKCC1 Expression during Neuronal Development

The developmental shift in GABA_A_R-mediated responses from depolarizing to hyperpolarizing was initially suggested to be due to postnatal downregulation of NKCC1 expression [134]. However, this shift was later shown to be the result of developmental upregulation of the Cl^−^-extruding transporter KCC2 [4]. KCC2 mRNA and protein levels are strongly upregulated in the brain during the second postnatal week in rats [4,5,159] and around the time of full-term birth in humans [5,8,9]. However, there is still no consensus on whether KCC2 upregulation is accompanied by a concurrent NKCC1 downregulation, or whether NKCC1 mRNA and protein levels remain high in adult neurons, and whether the expression profile differs between individual neuronal populations.

In the present context, it is worth noting that downregulation of NKCC1 functionality during neuronal development (as detected, for instance, in electrophysiological experiments on E_GABA_) does not necessarily require downregulation of the total amount of the NKCC1 protein, measured as number of molecules per cell. This is because cell input impedance, as well as, in parallel, ionic trafficking via channels and pumps, increases drastically as the neuronal soma and especially the dendritic tree grows. In other words, a given number of NKCC1 (or other transporter) molecules would have a much higher functional impact on [Cl]_i_ in a small electrically tight immature neuron than in a mature one. This kind of consideration is usually not taken into account when discussing neuronal development and the expression levels of ion transport proteins. Another important corollary is that even a small number of transporter molecules may well have a functional impact in a small subcellular compartment, such as the axon initial segment. Thus, interesting questions for future research concern the extent to which developmental changes in, e.g., GABA_A_R responses reflect quantitative shifts in the levels of Cl^−^-transporting proteins, and the extent of their altered targeting and localization within a given neuron. An additional factor to be taken into account is the post-translational modifications, such as the phosphorylation state of the CCCs, a topic that has been reviewed elsewhere [140,141,160,161].

Considering the difficulties in NKCC1 detection in adult animals, it is hardly surprising that the literature regarding the developmental pattern of NKCC1 mRNA and protein expression is, once again, highly contradictory. In the human and rodent forebrain, several studies have reported a robust developmental upregulation [8,70,72,75,133,162], while a similar number of studies observed a clear downregulation [68,71,73,134,163,164,165,166]. Corresponding discrepancies are seen in many neuronal structures [65,69,132,135,136,137,164,167,168,169,170], and the contradictory results do not seem to be easily explained on the basis of animal species (mouse, rat, or human), developmental stages, brain areas, methods of analysis, or probes used for detection (Table 1). In addition to the possible sources of variability and technical flaws listed above, there are other issues that could arise when brain sections from immature and mature animals are processed in parallel for immunohistochemistry or in situ hybridization analysis. For instance, due to the high lipid content of the mature compared to the immature brain, paraformaldehyde fixation may result in different crosslinking density, leading to varying accessibility of mRNA and protein targets to their corresponding probes and antibodies. In the case of in situ hybridization, one more putative factor accounting for the observed data inconsistency is the protease treatment required for efficient probe binding to the target mRNAs. When brain sections are prepared using animals of different ages and processed in parallel for in situ hybridization, the same protease treatment may not be optimal for NKCC1 mRNA detection in the immature and adult brain samples, thus distorting quantitative comparisons.

## 8. Implications for Future Work

As a conclusion, there is no consensus in the existing literature—even at the qualitative level—on the expression levels of NKCC1 in neurons and glia in the different regions of the developing and mature CNS. However, as stated above, the data available in single-cell RNAseq databases indicate that, in general, NKCC1 expression in glia is much higher than in neurons.

The expression of NKCC1 in various cell types of the brain makes it hard to quantify the mRNA and protein levels and changes therein in a meaningful manner. Indeed, when NKCC1 mRNA or protein levels are assessed en masse from large brain areas by RT-PCR or Western blot, the signal originates from mixed cellular populations, including not only glia but also the vasculature (pericytes and smooth muscle cells), thus preventing any quantitative assessment of neuronal NKCC1 levels. Nevertheless, data of this kind have been published in a large number of papers, which is the major reason for the existing confusions and contradictions. The broad cellular expression patterns of NKCC1, which contrast with the neuron specificity of KCC2, make calculating a ratio between KCC2 and NKCC1 mRNA or protein in brain tissue samples utterly meaningless.

Thus, single-cell approaches are an absolute necessity in future NKCC1 research. In addition to the single-cell RNAseq, single-molecule fluorescence in situ hybridization (smFISH) is a powerful technique to study NKCC1 expression in the brain with subcellular resolution and single-mRNA molecule sensitivity, and the various multiplexed variants of the technique enable simultaneous detection of up to thousands of different mRNA targets in the same cell [171,172]. At the protein level, commercially available scWestern systems allow simultaneous analysis of approximately 1000 cells in one run and quantification of about a dozen different proteins per cell [131]. The efficiency of the scWestern method has recently been demonstrated in a study in which the protein expression of KCC2 was examined in single arginine vasopressin neurons in the supraoptic nucleus of the hypothalamus [173].

A further important task is to gain information on the localization of the NKCC1 protein in specific subcellular compartments. Using conventional immunohistochemistry in studies on NKCC1 has not been successful in the CNS. This is mainly due to the lack of selective antibodies for work in CNS tissue. A systematic search for the optimal epitopes by high-throughput screening might help in designing novel antibodies. The recently published 3D structures of NKCC1 [23,24] and the increasing knowledge on the properties of relevant sub-molecular domains should also prove useful for the identification of suitable epitopes. Most importantly, and regardless of the approach, the selective immunoreactivity should be scrupulously controlled using corresponding tissue from a knock-out animal.

An alternative approach for labeling endogenous NKCC1 is genetic tagging. While overexpression of fluorescently tagged protein can cause mislocalization due to the exorbitant expression levels of exogenous promoters, knock-in strategies targeting the endogenous genomic locus present a better approach. For instance, clustered regularly interspaced short palindromic repeats (CRISPR)-Cas9-mediated homology-directed repair has been successfully used in combination with in utero electroporation for several endogenous proteins, such as CaMKIIα and CaMKIIβ, the two major subunits of the Ca^2+^/CaM-dependent kinase II (CaMKII), to visualize their localization in single neurons in vivo [174].

To conclude, the striking inconsistencies in the published literature regarding NKCC1 expression and localization must be readdressed with more reliable single-cell methods. Only then can we identify and experimentally approach the urgent questions on the roles of NKCC1 in brain functions, development, and pathology.

## Figures and Tables

**Table 1:** Comparison of the literature on developmental regulation of NKCC1.

Study	Journal	Species	Structure	Age Range	Method	Target Region	Knock-Out Control ^1^	Cell Types Expressing NKCC1	
								Earliest Age Point	Latest Age Point
**Studies Reporting an Upregulation of NKCC1 During Development:**									
Sedmak, 2015	*Cereb Cortex*	Human	Ctx, Hc, Th, Str, Cb	GW11–90Y	Microarray	Entire gene	-	-	-
Morita, 2014	*J Neurosci*	Human	Ctx	GW14–78Y	PCR	(21)	-	-	-
Hyde, 2011	*J. Neurosci.*	Human	Ctx, Hc	GW14–80Y	PCR	(17)	-	-	-
Li, 2008	*Vis. Neurosci.*	Mouse	Ret	P0–P20	IHC	(6)	Yes	Inner plexiform layer, neuroplastic cells	Horizontal cells, rod-bipolar cells, photoreceptors
Zhang, 2007	*J. Neurophysiol.*	Mouse	Ret	P0–P90	WB, IHC	(7)	Yes	Müller cells	Horizontal cells
Balakrishnan, 2003	*J. Neurosci.*	Rat, Mouse	Bs	P3–P12	ISH, PCR	(12) (25)	-	None	Lateral superior olive neurons
Mikawa, 2002	*Dev. Brain Res.*	Rat	Cb	P1–P49	ISH	(19)	-	None	Granule cells, glia
Wang, 2002	*Dev. Brain Res.*	Rat	Hc, Th	E18–P40	ISH	(19)	-	Neuroepithelium (Hc)	Neurons (Hc)
Yan, 2001	*Brain Res.*	Rat	Ctx, Hc, Cb, Th, Str	P0–Adult	WB, IHC	(6)	-	Neurons	Neurons (Ctx, Hc, Cb, Th), astrocytes (Ctx, Hc, Cb)
Sun and Murali, 1999	*J. Neurophysiol.*	Rat	Ctx	P0–P9	WB	(6)	-	-	-
**Studies Reporting a Downregulation of NKCC1 during Development:**									
Liu and Wong-Riley, 2012	*Neuroscience*	Rat	Bs	P0–P21	IHC	(6)	Yes	Neurons	Neurons, glia
Lee, 2010	*J. Neurochem.*	Rat	Ctx	P5–P90	PCR	(18)	-	-	-
Stil, 2009	*Neuroscience*	Rat	Sc	E17–P20	IHC	(6)	-	-	-
Delpy, 2008	*J. Physiol.*	Mouse	Sc	E11.5–P0	IHC	(6)	-	Motoneurons	Motoneurons
Aronica, 2007	*Neuroscience*	Human	Ctx	2M–30Y	IHC	(8)	-	Neurons	Neurons, astrocytes
Dzhala, 2005	*Nat. Med.*	Rat, Human	Ctx	P3–Adult, GW19–5Y^2^	WB, IHC	(8)	-	Neurons, non-neuronal cells	Neurons, non-neuronal cells
Yamada, 2004	*J. Physiol.*	Rat	Ctx	P1–P20	PCR	(16)	-	Cortical plate neurons	-
Ikeda, 2003	*Brain Res.*	Rat	Ctx	P0–P28	ISH	(19)	-	Neurons	Neurons
Shimizu-Okabe, 2002	*Neuroreport*	Rat	Ctx	P1–P28	ISH	(19) (20)	-	-	-
Wang, 2002	*Dev. Brain Res.*	Rat	Ctx	E18–P40	ISH	(19)	-	Neuroepithelium, cortical plate	Neurons, glia
Hübner, 2001	*Mech. Dev.*	Mouse	Ctx, Hc	E12.5–Adult	ISH	(10)	-	-	-
Plotkin, 1997	*J. Neurobiol.*	Rat	Ctx, Hc, Cb	P0 Adult	WB, IHC, ISH	(7)	-	Neurons, transiently at P14 Oligodendrocytes	Neurons
**Studies reporting stable expression of NKCC1 during development:**									
Mao, 2012	*J. Neurophysiol.*	Rat, Mouse	DRG	P1–Adult	PCR, IHC, ISH	(2) (5) (6) (7) (13) (23) (24)	Yes (IHC)	Neurons	Neurons
Ikeda, 2003	*Brain Res.*	Rat	Th	P0–P28	ISH	(19)	-	Neurons	Neurons
Marty, 2002	*Eur. J. Neurosci.*	Rat	Hc	P1–P60	IHC	(6)	-	Neurons	Pyramidal neurons, interneurons

^1^ Knock-out (KO) control shown for the particular staining paradigm. ^2^ Peak of expression GW 34. Bs = brainstem, Cb = cerebellum, Ctx = cerebral cortex, DRG = dorsal root ganglion, Hc = hippocampus, Ret = retina, Sc = spinal cord, Str = striatum, Th = thalamus, GW = gestational week.
